# Augmenting clozapine with other antipsychotics: results from two nationwide cohorts

**DOI:** 10.1192/j.eurpsy.2025.2273

**Published:** 2025-08-26

**Authors:** J. Tiihonen, A. Tanskanen, E. Mittendorfer-Rutz, H. Taipale

**Affiliations:** 1 Department of Forensic Psychiatry, Niuvanniemi Hospital, University of Eastern Finland, Kuopio, Finland; 2 Department of Clinical Neuroscience, Karolinska Institutet, Stockholm, Sweden; 3 Neuroscience Center, University of Helsinki; 4 Population Health Unit, Finnish Institute for Health and Welfare, Helsinki, Finland

## Abstract

**Introduction:**

A large proportion of patients with schizophrenia do not have a sufficient response even to clozapine. Very little is known if any pharmacological augmentation treatment can improve the long-term outcome of these patients.

**Objectives:**

We studied the comparative effectiveness of oral risperidone, olanzapine, quetiapine, and aripiprazole augmentation of clozapine treatment on the risk of hospitalization due to psychotic episode as a marker for severe relapse among patients with schizophrenia.

**Methods:**

In this population-based study, patients with schizophrenia or schizoaffective disorder using clozapine were included from Finnish (years 1996-2017) and Swedish (years 2006-2021) nationwide registers of inpatients care, specialized outpatient care, sickness absence, and disability pension. The risk of hospitalization associated with periods of antipsychotic augmentation vs. clozapine monotherapy (expressed as adjusted hazard ratio, aHR) was assessed by a within-individual design, using each individual as his/her own control, and analyzed with stratified Cox models. The two national cohorts were first analyzed separately, and then results were combined using a random-effect meta-analysis. Secondary outcomes were somatic hospitalization and composite outcome of psychosis/ somatic hospitalization.

**Results:**

In the meta-analysis of 23,206 clozapine users, medium dose (9-16.5 mg/day) aripiprazole augmentation was associated with the lowest risk of relapse among patients with low-dose (< 180 mg/day) (meta-analysis aHR 0.67, 95% CI 0.46-0.97, p=0.03), medium-dose (180-330 mg) (0.79, 0.70-0.91, p= 0.0006), and high-dose (>330 mg) clozapine (0.68, 0.62-0.75, p<0.0001), compared with the same clozapine dose as monotherapy. Augmentation with higher dose of aripiprazole or with other antipsychotics was associated with less favorable outcome. Only aripiprazole augmentations were associated with decreased risk of psychosis/somatic hospitalization, and the lowest risk was observed for medium-dose aripiprazole plus high-dose clozapine (0.70, 0.58-0.84, p=0.0001). Medium-dose aripiprazole plus high-dose clozapine was not associated with the risk of somatic hospitalization (0.66, 0.30-1.44, p=0.29), when compared with clozapine monotherapy in the same dose category.

**Conclusions:**

This meta-analysis of two nation-wide cohorts totaling over 23,000 clozapine using patients indicates that 10-15 mg/day aripiprazole augmentation of clozapine treatment is associated with about 20-30% decreased risk of relapse compared with clozapine monotherapy periods within the same individuals.

**Disclosure of Interest:**

J. Tiihonen Grant / Research support from: Janssen, Consultant of: Healthcare Global Village, HLS Therapeutics, Janssen, Orion, Teva, WebMD Global, Speakers bureau of: Janssen, Lundbeck, Otsuka, A. Tanskanen Grant / Research support from: Janssen, E. Mittendorfer-Rutz Grant / Research support from: Janssen, H. Taipale Grant / Research support from: Janssen, Speakers bureau of: Gedeon Richter, Lundbeck, Otsuka

